# Factors associated with non-adherence during tuberculosis treatment among patients treated with DOTS strategy in Jayapura, Papua Province, Indonesia

**DOI:** 10.1080/16549716.2018.1510592

**Published:** 2018-11-05

**Authors:** Yacob Ruru, Mariana Matasik, Antonius Oktavian, Rosliana Senyorita, Yunita Mirino, Lukman Hakim Tarigan, Marieke J. van der Werf, Edine Tiemersma, Bachti Alisjahbana

**Affiliations:** a Faculty of Public Health, Cenderawasih University, Jayapura, Indonesia; b Papua Provincial Health Department, Jayapura, Indonesia; c Institute of Research and Development for Biomedicine, Jayapura, Indonesia; d Sentani Public Health Center, Jayapura, Indonesia; e Faculty of Public Health, University of Indonesia, Depok, Indonesia; f European Center for Diseases Prevention and Control (ECDC), Solna, Sweden; g KNCV Tuberculosis Foundation, The Hague, The Netherlands; h Faculty of Medicine, Universitas Padjadjaran, Dr. Hasan Sadikin General Hospital, Bandung, Indonesia

**Keywords:** Tuberculosis, DOTS, case-control study, non-adherence, lost to follow up, Papua

## Abstract

**Background**: Despite the implementation of Directly Observed Treatment Short-course (DOTS) strategy in all public health centers in Papua Province, Indonesia, since 1998, the rate of loss to follow-up (LTFU) during tuberculosis (TB) treatment remains high (above 16%).

**Objectives**: We aimed to identify factors associated with non-adherence during TB treatment among patients treated at public health centers (PHCs) in Jayapura, Papua.

**Method**: We conducted a case-control study including new TB patients registered at eight PHCs from 2007 to 2009. Non-adherent cases were TB patients with a history of not taking anti-TB drugs for >2 consecutive weeks or >30 days cumulatively. Controls were randomly selected from patients who completed all doses of TB drugs in time. Data were collected by face-to-face interview using a pre-structured questionnaire and analyzed with logistic regression models.

**Results**: Data were available for 81 of 103 eligible cases and 183 of 206 eligible controls. Difficult access to healthcare (i.e. reported to have a problem with distance/travel cost and history of moving residence in the past year), lack of TB knowledge (i.e. lack of knowledge about TB transmission and the cause of TB and unawareness of the consequences of stopping TB treatment), and treatment experience (i.e. lack of TB education provided by TB nurse and the use of loose vs. fixed-dose combinations) were associated with non-adherence during TB treatment in the adjusted model, as were being aged under 35 years and having a history of TB in the family.

**Conclusion**: Our results suggest the need to improve TB treatment delivery especially to those who have difficult access to healthcare, and to routinely provide education to increase patients’ knowledge about TB and TB treatment. In addition, more attention to younger patients and those with a history of TB in their family is also needed.

## Background

Tuberculosis (TB) is an infectious disease that remains a public health problem in the world. Complete cure requires 6 months of treatment without interruption with multiple drugs which is challenging for patients and health care workers. Incomplete TB treatment may cause prolonged TB transmission, increased risk of development of drug-resistant TB, and higher mortality [1]. The need for good adherence to TB treatment has been acknowledged and emphasized by the WHO/IUATLD strategy known as DOTS [2,3].

**Figure 1.**
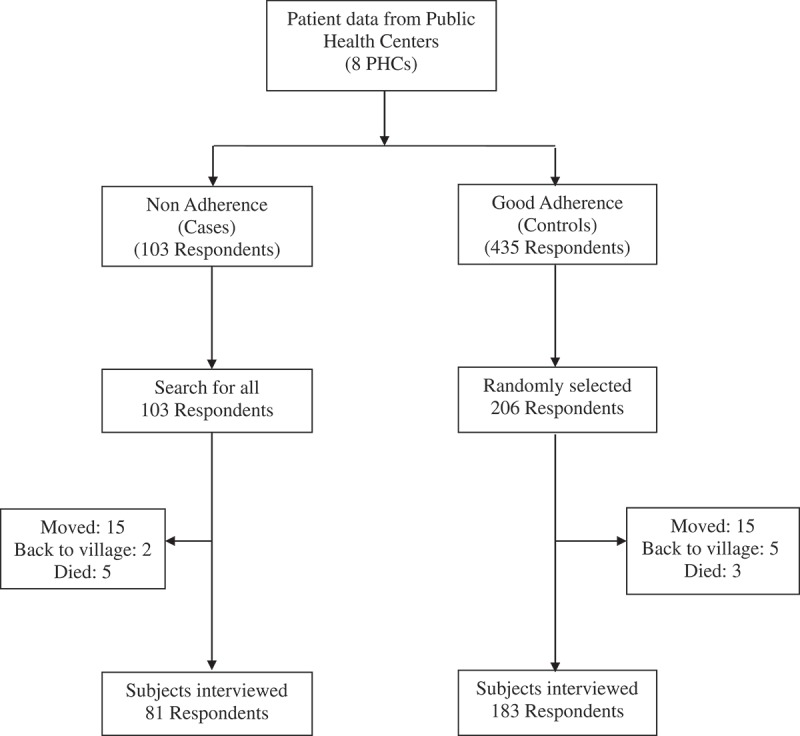
Sampling process.

To meet the World Health Organization (WHO) target to end TB by 2035, Indonesia has adopted a plan to stop TB which includes progressive control activities. DOTS has been implemented in the country since 1996, however it was estimated that in 2016, only 35% of the estimated total number of sputum acid-fast bacilli positive patients in Indonesia was notified, while the cure rate was 86% [4]. In the global setting, Indonesia has the second highest TB burden in the world with an estimated 1 million TB cases per year, or 391 incident TB patients per 100,000 population per year [4]. Among newly and previously treated TB cases, 2.8% and 16% are estimated to have multidrug-resistant (MDR) TB respectively [4]. The estimated HIV prevalence among TB patients in Indonesia is 0.4% [4].

Indonesia has 33 provinces and consists of thousands of islands. Papua is the easternmost province in Indonesia and has the lowest population density (10 inhabitants/km^2^). The population is scattered and there are limited transport facilities [5]. In 2016 the Papua province TB control program notified 9522 TB cases (case notification rate of 302.4/100,000 population) [6]. The proportion of TB cases with MDR-TB was estimated at 2% among primary cases in Papua [7,8]. The provincial TB program identified 42 MDR-TB cases among all cases notified in 2016. Papua province has the highest HIV prevalence in Indonesia [9]. Thirteen percent of TB patients diagnosed in 2009 were reported to be co-infected with HIV [10]. TB treatment success reached 70.0% in 2015 and was 64.4% in 2016. The low success rate is partly due to a high loss to follow up (LTFU), 16.4% in 2016 [11].

Jayapura, the capital city of Papua, is a rapidly expanding urban community with a quite well-developed infrastructure in comparison to other parts of Papua. The TB treatment success rate in Jayapura was low when this study was conducted (57.3%) [12] and was still low in 2016 (67.9%). This persistently low success rate was likely due to non-adherence and LTFU [6], but reasons for the low success rate have never been investigated in Papua. However, predictors of non-adherence to TB treatment have been identified in several reports from other parts of Indonesia. For example, a study conducted in a specialized lung clinic in Bandung (West Java, Indonesia) found that inadequate financial resources, transport to healthcare, lack of family support, and dissatisfaction with health care workers were associated with non-adherence [13]. Two qualitative studies conducted in Central Java identified feeling healthy, lack of knowledge about TB and its treatment, and changing health care facility during the course of TB treatment as independent risk factors for non-adherence during TB treatment [14,15].

In 2009, a study aiming to determine factors associated with treatment non-adherence among patients with TB in Jayapura City was done. Since then, no evidence has become available while the problem of low treatment success rates persists. We herewith present the 2009 study to inform the provincial and national TB program as well as countries facing similar problems with sub-populations about risk factors and potential interventions to increase treatment adherence among TB patients in Papua.

## Methods

### Study setting

We conducted our study in Jayapura city which has a population of 283 thousand [6]. It is served by 6 hospitals and 12 Public Health Centers (PHCs) besides an unknown number of private practitioners. TB patients may initially be diagnosed in hospitals, PHCs or other health centers by their symptoms and confirmed by sputum smear microscopy. Subsequently, most patients will be registered and receive their treatment at PHCs. At the PHC, TB management relies heavily on TB nurses. TB nurses register new TB patients, perform home visits during the first week of treatment, and follow patients during their treatment at the PHC. Monitoring of drug taking is done by a treatment observer, which in most cases is a family member of the TB patient [6]. The TB nurse provides training to the TB patient and the treatment observer at the beginning of a treatment program. Patients, with their treatment observer, are expected to come to the PHC every month to get their medication from the TB nurse. If a patient fails to come to the PHC, the TB nurses will call the patient or the family and visit the patient’s home if receiving no response by telephone. The use of fixed-dose combination (FDC) drugs was first introduced in 2008. Our study was conducted during the transition period from loose drug regimens to the fixed drug combination (FDC).

### Study design

We conducted an unmatched case-control study from January 2007 to January 2009. We defined cases as patients who were more than two weeks late in picking up their TB drugs on at least one occasion, or whose delayed visits for picking up the TB drugs accumulated to more than 30 days overall at the end of treatment. As per WHO definition, a patient whose treatment is interrupted for two consecutive months or more is regarded as lost to follow up (LTFU) [16] and consequently, these patients were automatically included as cases. We defined controls as patients who had completed the treatment with no history of non-adherence as described above. To obtain our study sample, we first compiled a list of eligible adult patients (i.e. ≥15 years old) from eight PHCs in Jayapura City (Tanjung Ria, Imbi, North Jayapura, Hamadi, Elly Uyo, Kotaraja, Hedam, and Waena) who started their TB treatment between 1 January 2007 and 31 March 2008. The other five PHCs in the city were purposively excluded in our study for logistical reasons. No sample size calculation was done; all eligible patients meeting the case definition were approached and invited to participate in our study. Controls were selected from the same PHCs. We randomly selected twice as many controls as cases (2n).

### Definition and measure

Demographic data, initial sputum acid-fast bacilli result, TB medication pickup record, and HIV status were extracted from the TB treatment record card (referred as the TB1 card in Indonesia’s NTP Guidelines) [3]. All eligible cases and controls were contacted for an interview by a trained interviewer using a pre-structured questionnaire. We conducted the interviews between 1 July 2008 and 31 January 2009. Information collected included sex, age, ethnicity, household income, level of education, and household density. We classified ethnicity as ‘Papuan’ and ‘non-Papuan’. Household income was recorded and presented in million Indonesian Rupiah (IDR) per month. Education level was classified in two categories, ‘no formal education or did not finish primary school’ and ‘graduated from primary school or higher’. Household density was calculated by dividing the number of people living in the household by the estimated size of the house (in square meters). We defined overcrowding as more than one person living on 10 m^2^. We collected data about treatment experience, including (a) TB education received from the TB nurse, (b) type of drugs (i.e. loose drugs vs. FDC), (c) having a treatment observer, and d) the presence of any adverse drug reaction during TB treatment. Information regarding mobility and access to a health care center were collected by asking patients about (a) any history of moving residence within the last year, (b) distance to PHC, (c) perceived barriers to go to PHC (i.e. is it considered difficult or expensive to get to the PHC to collect the medication). Knowledge of TB was measured by asking TB patients about: (a) the cause of TB disease, (b) how TB is transmitted, (c) curability of TB disease, (d) duration of a full TB treatment course, and (e) consequences of incomplete TB treatment. Risk of alcoholism was assessed with the TWEAK questionnaire [17]. We assessed the patients’ general physical condition using the Karnofsky’s score [18].

### Statistical analysis

Data were entered in EpiData v2.1 and analyzed using SPSS 19.0 (IBM Corporation). For categorical predictors, differences in proportions were tested for statistical significance using Chi-square or Fisher’s exact test when appropriate. Differences in continuous predictors were tested using the Mann-Whitney test. All factors associated with non-adherence (Wald *X^2^* p-value < 0.10) at the bivariate level were included in the multivariable logistic regression model to calculate the adjusted ORs (aORs) and 95% Confidence Intervals (CI). The final multivariable logistic regression model was fitted using the forward selection method. The likelihood ratio test was used to assess goodness of fit of the final model. Sensitivity analysis was performed to account for potential bias due to non-response. For the sensitivity analysis, patients (i.e. cases and controls) included in our final analyses were compared to non-responders with respect to their basic demographic characteristics.

## Results

Between January 2007 and March 2008, there were 548 TB patients registered in the eight participating PHCs in Jayapura. Of these, 103 (18.8%) met the case definition criteria, and 435 were classified as controls. Of the 103 cases, we were able to interview 81 (78.6%). Twenty-two cases were not included in our study as they either no longer lived at the address listed in the TB register (n = 17) or had died (n = 5). Of the 435 controls, 206 were randomly selected, and 183 (88.8%) of those were interviewed. Twenty-three controls could not be interviewed because they either had been transferred to other health centers (n = 15), had returned to their home village (n = 5), or had died (n = 3). Among those contacted, all agreed to be interviewed (Figure 1). We did not observe differences in age and gender distribution among cases and controls included in our final analysis and non responders (Supplementary Table 1)

Seventy-two (88.9%) cases terminated their treatment during the initiation phase (i.e. first two months of TB treatment). Of the 9 cases who completed the treatment, TB treatment was finalized within a median of 7.3 months (IQR 7.0–7.5 months). Controls completed their treatment within a median of 6 months (IQR 5.8–6.3 months). Cases were significantly younger than controls (median age 26 years (IQR 22–35) vs. 32 years (IQR 24–40) (p = 0.009)). The proportion of females among cases was comparable to the proportion among controls, i.e. 46.9% and 38.8% respectively (p = 0.22). There was no statistical difference in the proportion of acid-fast bacilli positive TB among cases (54/81, 66.7%) and controls (127/183, 69.4%) (p = 0.70). Compared to controls, cases were more likely to be of Papuan ethnicity (p = 0.01), had a poorer health condition as indicated by a lower Karnofsky score (p < 0.005) and were more likely to have TB-related symptoms at the time of interview (p = 0.001). Cases were also more likely to be at risk of alcoholism (p = 0.022), and to have at least one member of their household ever treated for TB (p = 0.01) (Table 1).

Compared to controls, cases were more likely to receive loose TB drugs rather than FDC (p = 0.001). Cases also more frequently perceived barriers to access TB treatment (i.e. difficulties with travel to the treatment center and transportation costs) (p < 0.001). We observed that cases were less likely to receive an explanation about their disease from health workers (p = 0.007) and they had significantly lower knowledge of the five aspects of TB management compared to controls (Table 2). Also, cases were less likely to report side effects such as headache, nausea, vomiting, joint pain, and itching (p = 0.012).

**Table 1.** Treatment history, demographic and household characteristics associated with adherence during tuberculosis treatment among tuberculosis patients from Jayapura City.

	Cases (poor adherence) (n,%) (N = 81)	Controls (good adherence) (n,%) (N = 183)	p-value^§^
Basic & treatment characteristics			
Age in years, median (25th–75th percentile)	26 (22–35)	32 (24–40)	0.009^#^
Female sex	38 (46.9)	71 (38.8)	0.22
Sputum acid-fast bacilli positive at treatment initiation	54 (66.7)	127 (69.4)	0.70
Treatment outcome			<0.001
Cured	12 (14.8)	132 (72.1)	
Treatment completed	1 (1.2)	51 (27.9)	
Lost to follow up	68 (84.0)	0 (0.0)	
Findings from interview			
Place of interview			<0.001
In health center	32 (39.5)	121 (66.1)	
In respondent’s home	49 (60.5)	62 (33.9)	
General health			
Karnofsky score <90%* at interview	13 (16.0)	8 (3.3)	<0.001
Having TB-symptoms at interview^£^	54 (66.7)	80 (43.7)	0.001
At-risk for alcoholism^†^	12 (14.8)	10 (5.5)	0.022
Demographics			
Papuan ethnicity	60 (74.1)	105 (57.4)	0.01
No or low-level education only‡	12 (14.8)	34 (18.6)	0.57
Job type before tuberculosis			0.54
Unemployed	7 (8.6)	15 (8.2)	
Housewife	22 (27.2)	45 (24.6)	
Civil servant	4 (4.9)	18 (9.8)	
Self-employed, trading	16 (19.8)	42 (23.0)	
Student	17 (21.0)	25 (13.7)	
Other (incl. farmers, fishermen)	15 (18.5)	38 (2.8)	
Household factors			
Household income, median (25^th^–75^th^ percentile) in (×10^6^IDR)	1.2 (0.9–2.0)	1.5 (1.0–2.0)	0.42^#^
>1 Persons per 10m^2^ in house	17 (21.0)	49 (26.8)	0.14
Household member ever treated for TB	29 (35.8)	37 (20.2)	0.01

^§^p-values were calculated using chi-square test for proportions except the continuous variable using ^#^Mann-Whitney equality-of-populations rank test to test differences between medians.

*Karnofsky score to describe performance status (Oxford Textbook of Palliative Medicine, Oxford University Press, 1993). A score of 90 means has minor signs of disease, less than 90% shows more

signs of disease and unable to carry normal activity.

^£^The following TB symptoms were reported most frequently: coughing that last three or more weeks, coughing of blood, chest pain, pain with breathing or coughing, unintentional weight loss, fatigue, fever, night sweats, chills, loss of appetite

^†^At-risk for alcoholism was defined as a TWEAK-score of >2 [17].

^‡^Low-level education defined as no education or did not finish primary school.

IDR, Indonesian Rupiah

**Table 2.** Experience, perception, and knowledge about tuberculosis associated with adherence status among tuberculosis patients.

	Cases (poor adherence) (n,%) (N = 81)	Controls (good adherence) (n,%) (N = 183)	p-value^§^
Treatment experience			
Has not received education about TB from the TB nurse	12 (14.8)	8 (4.4)	0.007
Receiving loose drug rather than fix drug combination	12 (14.8)	7 (3.8)	0.001
Reports to have a treatment observer	57 (70.4)	138 (75.4)	0.48
Experiencing side effects during TB treatment*	33 (40.7)	45 (24.6)	0.012
Mobility and access			
Moved residence in the last year	9 (11.1)	3 (1.6)	0.001
Travel time >15 minutes to the public health center	15 (18.5)	32 (17.5)	0.97
Perceives that transport to health center was difficult due to distance or costs	10 (12.3)	2 (1.1)	<0.001
Retained job during TB treatment	60 (74.1)	148 (80.9)	0.28
Perceives that costs of TB treatment are too high	23 (28.4)	29 (15.8)	0.02
Patient’s knowledge about TB			
Does not know what causes TB	63 (77.8)	95 (51.9)	<0.001
Does not know how TB is transmitted	41 (50.6)	34 (18.6)	<0.001
Thinks that TB cannot be cured	9 (11.1)	0 (0.0)	<0.001
Unaware about the duration of a full TB treatment course	10 (12.3)	4 (2.2)	0.001
Unaware about consequences of incomplete TB treatment‡	15 (19.0)	6 (3.3)	<0.001

^§^p-values were calculated using chi-square tests for proportions.

*This concerns the following symptoms: feeling sick or dizzy, skin rashes, pin and needles, flu like symptoms.

‡ Two cases did not answer this question, and therefore, the percentages were calculated on a total of 79 cases and 183 controls (total population: 262).

In the multivariable logistic regression model, we found that younger age (aOR 3.1 95% CI 1.5–6.6), history of moving in the past year (aOR 9.6 95% CI 2.2–42.0), history of TB in the family (aOR 2.5 95% CI 1.2–5.2), having received loose drugs (aOR 3.8 95% CI 1.2–12.0), and perceived healthcare access barriers (aOR 10.2 95% CI 1.7–60.0) were independently associated with non-adherence during TB treatment (Table 3). Other factors related to non-adherence to TB treatment in the same model were lack of knowledge about three aspects of TB, including the cause of TB (aOR 2.4 95% CI 1.1–5.4), TB transmission (aOR 3.8 95% CI 1.8–7.8), and consequences of not completing TB treatment (aOR 10.3 95% CI 3.1–34.0).

**Table 3.** Univariable and multivariable odds ratios (OR) and 95% confidence intervals (95% CI) for factors associated with non-adherence to tuberculosis (TB) treatment^‡^.

	Univariable				Multivariable^‡^			
Risk factors	OR	95% CI			aOR*	95% CI		
Individual characteristic								
Age < 35yrs	2.2	1.2	-	3.9	3.1	1.5	-	6.6
Papuan ethnicity	2.1	1.2	-	3.8	NS			
At risk for alcoholism	3.0	1.2	-	7.3	NS			
Family characteristics								
>1 person per 10m^2^ in household	2.0	1.1	-	3.4	NS			
Having a household member treated for TB	2.2	1.2	-	3.9	2.5	1.2	-	5.2
Access and mobility								
Moved residence in the last year	7.5	2.0	-	28	9.6	2.2	-	42
Distance/travel costs perceived as problem	12.7	2.7	-	60	10.2	1.7	-	60
Finds costs of TB treatment too high	2.1	1.1	-	3.9	NS			
Treatment experience								
Received loose drugs (instead of fixed dose combination)	4.4	1.7	-	12	3.8	1.2	-	12
Having side effect from TB treatment	2.1	1.2	-	3.7	NS			
Has not received education about TB	3.8	1.5	-	9.7	3.5	1.0	-	12
Patient knowledge								
Does not know the cause of TB	3.2	1.8	-	5.9	2.4	1.1	-	5.4
Does not know how TB is transmitted	4.5	2.5	-	8.0	3.8	1.8	-	7.8
Thinks that TB cannot be cured	3.5	2.9	-	4.3				
Unaware about the duration of a full TB treatment course	6.3	1.9	-	21	NS			
Unaware about the consequences of incomplete TB treatment	6.9	2.6	-	19	10.3	3.1	-	34

^‡^NS: not statistically significant in multivariable model (applying backward stepwise logistic regression; retaining all variables with a p-value of <0.05 in the model)

*Adjusted for all other variables with an adjusted OR in this table.

^‡^The total subject analyzed for the final model were 262 (79/81 cases, and 183/183 controls).

## Discussion

Our study identified several factors related to non-adherence to TB treatment among patients treated in PHCs in Jayapura. We found that younger age, a family history of TB treatment, difficulties in accessing healthcare (i.e. distance/cost), and a history of moving residence in the past year were independent risk factors for non-adherence during TB treatment. In addition, treatment experience including the use of loose drugs vs. FDC, having received any education from TB nurses, and TB knowledge (cause of TB, how TB is transmitted, and the consequence of incomplete TB treatment) were also significant risk factors associated with poor adherence or LTFU.

Among cases, LTFU and non-adherence during TB treatment occurred mostly during the intensive phase. Defaulting early in the treatment period has also been found in two studies from Bandung [13] and Mumbai [19]. In contrast, two studies conducted in Morocco and Southern Ethiopia observed that most LTFU occurred during the continuation phase [7,20]. The difference between these studies lies in the fact that in the two African studies drug-taking was observed directly by healthcare workers during the intensive phase, whereas in Indonesian settings, TB treatment was most frequently supervised by a family member and not by healthcare workers [10,15]. This observation questions the effectiveness of family members as drug observer.

The association between age and non-adherence during TB treatment has been investigated in other studies, with different results. Similar to our study, two African studies found that younger age is associated with non-adherence [20,21]. This association was also observed in the Indian and Moroccan studies [7,19]. Other studies showed that older age was significantly related to the risk of LTFU [22,23]. The studies in Bandung, Indonesia and Sub-Saharan Africa did not find any difference between age groups [13,24]. In our setting young people may face more substantial problems in coping with TB treatment in comparison to the older age groups or receive less support from their social network. This thus needs attention from the TB program.

Our analysis showed that a history of moving residence is an independent risk factor for poor adherence. This finding is supported by a large case-control study conducted by Finlay et al. [21] in South Africa. Other studies in Africa also show that difficulties of physical access and transfer of cases to other healthcare units related to moving of residence affected adherence to TB treatment negatively [20,24]. Jayapura as the capital city of Papua Province acts as the center for economic activities. The city experiences high urbanization from surrounding rural areas for seasonal work. Consequently, the proportion of people living in a temporary residence is high. This may explain the high proportion of patients with a history of moving residence in our data.

TB patients who perceived distance and travel cost as a problem were less likely to adhere to TB treatment in our study. Challenges with accessing healthcare due to distance or cost for transportation have been reported by several studies as a risk factor for non-adherence and LTFU during TB treatment [13,20,24–26]. Jayapura is the most developed municipality in Papua. However, public transportation and infrastructure within the city is still limited due to Jayapura’s geographical features. For instance, a large part of the city perimeter is mountainous and forested. Travel distance, geographical characteristics, and inadequate infrastructure make public transportation relatively expensive.

The majority of the participants had some knowledge about TB. However, their knowledge of three aspects, (i.e. the cause of TB, how TB is transmitted, and the consequence of incomplete TB treatment) was still low. Cases showed a significantly lower level of knowledge than controls on these three aspects. Previous studies examining risk factors for LTFU reported that low knowledge about the cause of TB, TB transmission and the consequences of incomplete treatment played a role in lower treatment compliance [14,19]. Many studies also demonstrated that inadequate knowledge of TB was associated with poor adherence [7,22–24,27,28]. In contrast to our finding, a similar case-control study conducted in urban Morocco found that TB knowledge (duration, causes, transmission, and consequence of stopping TB treatment) was not associated with compliance during TB treatment [7]. The fact that in this setting, patients had daily contact with a healthcare provider during observed treatment may explain this difference. A previous cohort study conducted in a different region in Indonesia (Bandung, West Java) also found no association between TB knowledge and LTFU [13]. This was probably due to the higher degree of patients’ awareness and knowledge about TB because treatment that was provided in a specialized lung clinic [13]. The level of TB knowledge of patients in our study was generally lower than patients’ knowledge in these two other studies.

Our study has several limitations. First, the time lag between the date of the end of the TB treatment and the time of interview (ranging from 0 to 24 months after stopping treatment) may have introduced recall bias. However, we cross-checked information gathered through the interview with the data available at the health center whenever possible to minimalize potential bias. Furthermore, the interval between the end of TB treatment and the time of interview was similar among cases and control suggesting that recall bias may have affected both groups similarly. Second, there is a possibility of patient misclassification (e.g. if days of missed doses were not recorded properly in the treatment card or register). Non-differential misclassification on adherence status may bias our estimates towards the null [29], however, we believe that outcome misclassification has much less impact than exposure misclassification in our analysis. Third, we were unable to assess all non-adherence risk factors investigated by other studies (e.g. the use of herbal medication and co-infection with HIV which could be relevant in the Papua setting) since that was operationally difficult at the time of data collection. Furthermore, we decided not to include any open-ended questions in our questionnaire to ease the data analysis process. Lastly, the study took place in the urban setting of Jayapura which by itself is not generalizable to another urban setting in other parts of Indonesia, nor to more rural settings in Papua Province.

## Conclusion

This study identified various risk factors associated with non-adherence during TB treatment, some of which can potentially be addressed through (targeted) interventions. From all investigated variables, variables that can be changed/intervened on include: (a) lack of information about TB from health staff; (b) low knowledge about the cause of TB, transmission of TB, and the effect of incomplete TB treatment. Intensified health promotion to the community and routine education of the patient by healthcare staff might overcome this problem. Adherence to treatment was positively affected by using FDC instead of loose drugs. FDC has now replaced loose drugs everywhere in Indonesia.

Some risk factors for non-adherence to TB treatment, such as younger age, history of moving in the past year, and living far from treatment centers, should signal public health officers of the possibility of non-compliance. These factors can be addressed by providing socio-economic support to patients (e.g. patient support packages [including transportation allowance] for patients with low household incomes or those who have to travel a long distance to the healthcare centers).

To ensure adherence among patients that are migrating, healthcare workers can provide better TB education on the importance of continuing TB treatment and the possibility to continue treatment at a different treatment center. The transfer of patients can also be assisted by an information system that can notify healthworkers on the whereabouts of patients during treatment transfer between hospital and primary health care and among primary health care institutions despite geographical difficulties.

## Paper context

Non-adherence or loss to follow-up rates are high in Papua. This publication presents factors related to non-adherence during TB treatment. Our finding suggests that health workers can design better treatment programs which provide TB patients with proper education about TB, identify which patients are at higher risk of non-adherence to TB treatment, and provide support for transportation. This study showed a positive effect of FDC on TB treatment adherence, which can support FDC implementation in the TB National Program.
