# Supplementary material for: Factors associated with non-adherence during tuberculosis treatment among patients treated with DOTS strategy in Jayapura, Papua Province, Indonesia
Source: Glob Health Action. 2018 Nov 5;11(1):1510592. doi: 10.1080/16549716.2018.1510592 (PMC6225439; doi:10.1080/16549716.2018.1510592)
Supplement: Supplemental Material [file ZGHA_A_1510592_SM6230.docx]

**Supplementary Tables**

**1. Sensitivity analysis**

To look for possible bias to the suboptimal response rate in cases and controls, we conduct a sensitivity analysis. These two tables describe the difference of the total case (and control) vs the responding case (and control) subjects

Table 1. Sensitivity analysis, diference responding subjects vs total subjects of **cases**

|  | total  subjects  (N=103) | responding subjects  (N=81) | nonresponding subjects  (N=22) | p-value  (total vs responding subjects) |
| --- | --- | --- | --- | --- |
| Basic & treatment characteristics |  |  |  |  |
| Age, median (25^th^-75^th^ percentile) | 28 (22 – 39) | 26 (22 – 35) | 31 (22,75 – 43,25) | 0,180 |
| Female sex | 50 (48,5) | 38 (46.9) | 12 (54,5) | 0,693 |

Table 2. Sensitivity analysis, diference responding subjects vs total subjects of **controls**

|  | total  subjects  (N=206) | responding subjects  (N=183) | nonresponding subjects  (N=23) | p-value  (total vs responding subjects) |
| --- | --- | --- | --- | --- |
| Basic & treatment characteristics |  |  |  |  |
| Age, median (25^th^-75^th^ percentile) | 30 (23 – 40) | 31 (24 – 40) | 25 (22 – 36) | 0,111 |
| Female sex | 75 (34,6) | 67 (36,3) | 8 (34,8) | 1,000 |

Conclusion: There is no difference of sex and age of the total respodent versus the subject analyzed in this paper.

**2. Differences of time lag to interview**

To look for possible bias due to time lag to interview, we compare the time lag from begining treatment and ending treatment to the interview, and compare the result between cases and control

Table. Lag time to interview from date of begining and ending of treatment among cases and control

|  | Cases  (n=81) | Control  (n=183) | P-Value |
| --- | --- | --- | --- |
| Time lag from begining of treatment in weeks (Median, IQR) | 44 (28 – 59) | 58 (32 – 69) | 0,007 |
| Time Lag from end of treatment in weeks (Median, IQR) | 28 (14-44) | 33 (6-43) | 0.186 |

Conclusion:

We found that there is a significant difference between cases and control in the time lag from the begining of treatment to interview. Cases are more likely to be interviewed earlier than control. This would be obvious as there are more LFUs among the cases and therefore being interviewer earlier.

On the contrary, we did not find significant difference in the time lag from end of treatment to interviews between cases and controls. Therefore, the bias due to time lag to interview would be considered minimal.
